# Under-estimation of maternal and perinatal mortality revealed by an enhanced surveillance system: enumerating all births and deaths in Pakistan

**DOI:** 10.1186/s12889-018-5363-3

**Published:** 2018-04-02

**Authors:** Jasim Anwar, Siranda Torvaldsen, Mohamud Sheikh, Richard Taylor

**Affiliations:** 1 School of Public Health and Community Medicine, the University of New South Wales, Sydney, Australia; 2 Department of Community Medicine, Women Medical and Dental College, Abbottabad, Pakistan; 3 Clinical and Population Perinatal Health Research, Kolling Institute, Sydney Medical School Northern, the University of Sydney, Sydney, New South Wales Australia

**Keywords:** Mortality surveillance, Maternal mortality, Perinatal mortality, Neonatal mortality, Stillbirths, Health information system, Health system strengthening, Pakistan

## Abstract

**Background:**

Reliable and timely data on maternal and neonatal mortality is required to implement health interventions, monitor progress, and evaluate health programs at national and sub-national levels. In most South Asian countries, including Pakistan, vital civil registration and health information systems are inadequate. The aim of this study is to determine accurate maternal and perinatal mortality through enhanced surveillance of births and deaths, compared with prior routinely collected data.

**Methods:**

An enhanced surveillance system was established that measured maternal, perinatal and neonatal mortality rates through more complete enumeration of births and deaths in a rural district of Pakistan. Data were collected over a period of 1 year (2015/16) from augmentation of the existing health information system covering public healthcare facilities (*n* = 19), and the community through 273 existing Lady Health Workers; and with the addition of private healthcare facilities (*n* = 10), and 73 additional Community Health Workers to cover a total study population of 368,454 consisting of 51,690 eligible women aged 18 to 49 years with 7580 pregnancies and 7273 live births over 1 year. Maternal, neonatal, perinatal and stillbirth rates and ratios were calculated, with comparisons to routine reporting from the previous period (2014–15).

**Results:**

Higher maternal mortality, perinatal mortality and neonatal mortality rates were observed through enhanced surveillance compared to mortality rates in the previous 1.5 years from the routine monitoring system from increased completeness and coverage. Maternal mortality was 247 compared to 180 per 100, 000 live births (*p* = 0.36), neonatal mortality 40 compared to 20 per 1, 000 live births (*p* < 0.001), and perinatal mortality 60 compared to 47 per 1000 live births (*p* < 0.001). All the mortality rates were higher than provincial and national estimates proffered by international agencies based on successive Pakistan Demographic and Health Surveys and projections.

**Conclusion:**

Extension of coverage and improvement in completeness through reconciliation of data from health information systems is possible and required to obtain accurate maternal, perinatal and neonatal mortality for assessment of health service interventions at a local level.

## Background

Pakistan is among the ten countries estimated to account for 60% of global maternal deaths. Based on interview survey methods, the Maternal Mortality Ratio (MMR) in Pakistan is estimated to have declined from 430/10^5^ live births in 1990 to 180 in 2015 [1], and neonatal mortality is estimated to have declined from 64/10^3^ live births in 1990 to 46/10^3^ live births in 2015 [2]. Similar to several other countries, Pakistan did not achieve the Millennium Development Goals (MDGs) 4 and 5 which relate to these indices. In 2015, countries adopted the 17 Sustainable Development Goals with 169 targets to be achieved by 2030 [3]. Of the 13 health targets, the first two are to reduce the estimated global maternal mortality ratio to less than 70/10^5^ live births, and reduce neonatal mortality (0–27 days) to ≤12/10^3^ live births [3]. In order to monitor the progress on these targets, a renewed emphasis has been placed on the need for reliable and timely data involving counting all births and deaths, especially around the time of birth [4].

Most of the available estimates of maternal and neonatal mortality rates from lower and middle-income countries have been reported at the national level, with a wide variation among countries [1, 2]. Pakistan is the sixth most populous country in the world with an estimated 185 million people in 2012–13 [5]. There are wide variations among indicators including mortality rates among the six provinces [6]. For example, the estimated MMR for Punjab province is 227/10^5^ live births, compared to 785 in Baluchistan [7], and the estimated Neonatal Mortality Rate (NMR) for Khyber Pakhtunkhwa province is 41/10^3^ compared to 63 in Punjab and Baluchistan [5]. In Pakistan, these indices are derived from demographic and health interview sample surveys (DHS), to estimate mortality rates at a national or provincial level, but provide no information on district or sub-district variations because of small sample sizes [5, 7].

The current routine health information systems in Pakistan that report data on pregnancies, births, and deaths are inadequate in several aspects. Neonatal mortality may be under-reported by the District Health Information System (DHIS), since it collects data only from public health facilities [8], excluding the 34% of the births in private health facilities [5].

The Lady Health Worker (LHW) Program operates at the community level but covers only 70% of the population. The LHWs register pregnant women, collect birth and death data, and provide family planning, health education and referral services to pregnant women and families in their areas. Some community level data collected by LHWs are not linked with the DHIS [9, 10]. This contributes to inadequate data available to decision makers [11]. Moreover, maternal deaths may be under-reported since LHWs follow pregnant women for medical risks only until parturition, whereas maternal mortality can occur up to 42 days after delivery. Maternal Newborn and Child Health Program data are not incorporated into the health facility reports, nor is their Program data linked with the DHIS [12, 13]. Thus there is no national or provincial health information system that reconciles data from all sources, including the private sector and community areas not covered by LHWs, to provide accurate maternal, perinatal and neonatal mortality rates at a district or sub-district level. The aims of this study were to: 1). test the feasibility of establishing an enhanced surveillance system that captures data from all available health information systems, and extends surveillance to areas without any information systems; 2). estimate maternal, perinatal and neonatal mortality rates by more complete enumeration of all pregnancies, births, maternal, perinatal, and neonatal deaths and derive estimates of under-enumeration by comparison with previous routinely collected data; 3). compare mortality rates calculated by the surveillance system with the national and sub-national mortality rates estimated by Demographic Health Surveys and international agencies.

## Methods

An enhanced surveillance system was established that endeavoured to capture all births and deaths using information from both public and private healthcare facilities, and extended community coverage, with an improved completeness of reporting and reconciliation of data.

### Study population

**Fig. 1:**
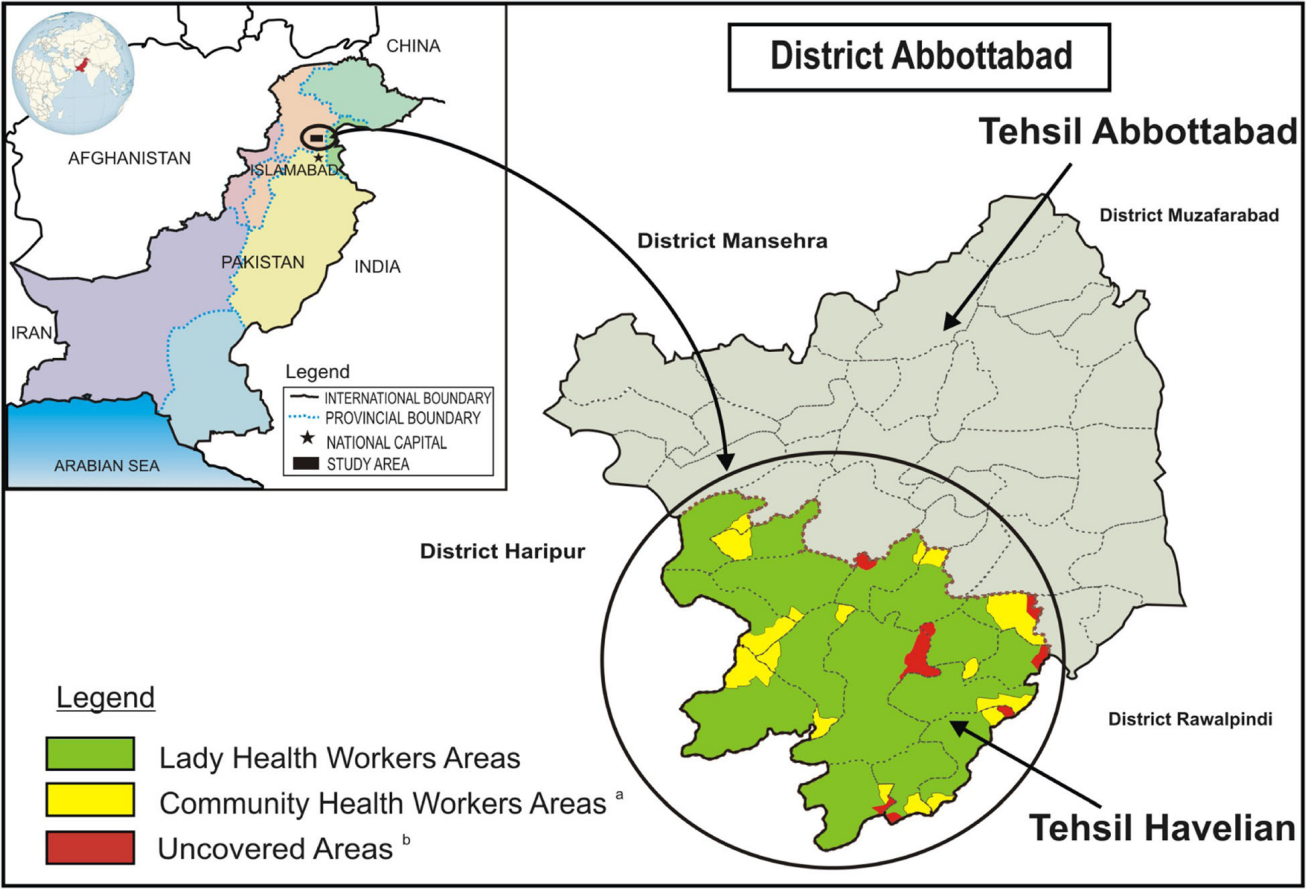
Map of study area, Tehsil Havelian, District Abbottabad, Pakistan. ^a^Extended coverage through Community Health Workers to previously uncovered areas. ^b^Community Health Workers were allocated to these areas, but did not stay for the entire duration of the study because of the arduous nature of the work in such remote locations. Uncovered areas comprise only 4% of the population of Tehsil Havelian

This population-based prospective study was conducted over 1 year (June 2015–May 2016) in Tehsil Havelian (a sub-district) of the District of Abbottabad of Khyber Pakhtunkhwa province located in the North of Pakistan, approximately 110 km from Islamabad (the capital of Pakistan). The estimated population of the District Abbottabad in 2010 was 1.179 million, and the study area of Tehsil Havelian was 341,891 (29% of the district population) [14]. Approximately 80% of the population of Tehsil Havelian live in rural areas, and 54% have completed primary level education [14, 15]. Figure 1 shows the geographical location of District Abbottabad and the study area. The total population (both sexes and all ages) of the study area registered by the LHWs and CHWs at the start of this study was 368,454, enumerated by visiting each household at the commencement of the study in June 2015. The difference between the estimated populations in 2010, and actual population in 2015, may be attributed to population growth. However, other factors, including in-migration related to effects of natural disasters, also contribute to the population growth. Of the total registered population, 293,344 (80%) resided in the LHWs areas and 75,110 (20%) resided in the areas of CHWs. The study period for enhancement of surveillance was from 1 June 2015 to 31 May 2016. The study population consisted of 51,690 married women aged 18–49 years who were permanent residents of Tehsil Havelian.

### Data sources and collection

Prior to the establishment of the enhanced surveillance system in the study area, data on pregnancies, births and deaths at the community (household) level were reported monthly by the LHWs to their program. Births and deaths that occurred at the community birth stations were reported by the Community Midwives. These monthly reports by LHWs and Community Midwives were entered at the District level and flow directly to their Provincial offices. Identified high-risk pregnancies by LHWs, were referred to appropriate hospitals but neither recorded, nor followed-up for the outcome. Births and deaths at public health facilities were reported to the District health office monthly and entered into the District Health Information System for onward submission to the Provincial DHIS office. These data, reported from the community or public health facilities, were not analyzed or used at District level for improving the maternal and child health. The DHIS lacks a mechanism to assign unique identifiers to the reported deaths.

**Fig. 2:**
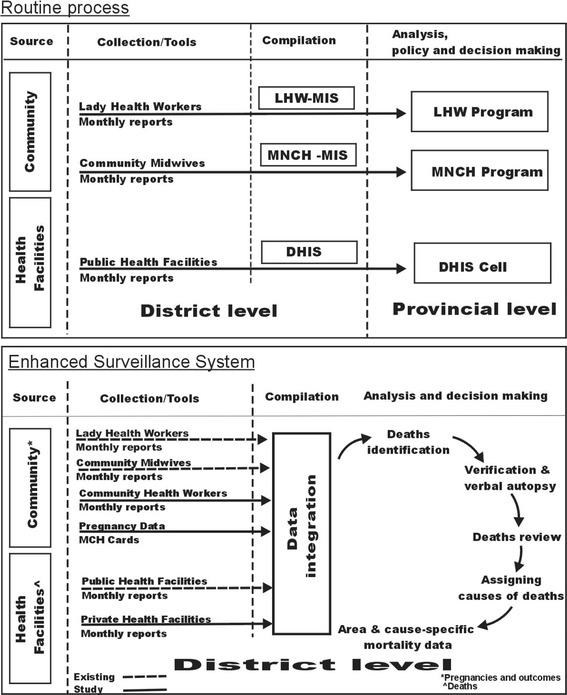
Birth and death reporting in Tehsil Havelian, District Abbottabad: existing routine reporting and the enhanced surveillance system. Abbreviations: LHW, Lady Health Worker; DHIS, District Health Information System; MIS, Management Information System

Following the establishment of the enhanced surveillance system in the study area pregnancies were recorded at the household level and followed after birth for maternal, perinatal and neonatal mortality by the LHWs. Monthly reports from the LHWs were aggregated at the community level by LHW supervisors. Community Health Workers were employed to record similar data from areas not covered by LHWs. Birth and death data were also captured from the private health facilities in addition to the routine monthly reports from public health facilities. Data from all sources (community level and health facilities) were integrated at the District office. All deaths (maternal, neonatal, early neonatal and stillbirths) were recorded from the community level (households), and from all health facilities (public and private), and verified directly from the households, if the death met the inclusion criteria. Figure 2 shows the routine process of data collection on births and deaths, and the extensions made to the routine process by the enhanced surveillance system.

A total of 283 LHWs were engaged in data collection that covered 79% of the study population in Tehsil Havelian, Abbottabad District. Each LHW provides services to a population of 800–1000 in a defined geographical area. LHWs maintain a register of all married women aged 18–49 years for their assigned households and visit 7–10 houses per day to update the records and registers. LHWs register any pregnant women they find during their routine visit to the households, and prepare a mother and child health card for follow-up. In an endeavour to capture all pregnancies, births and deaths at the community level, an additional 73 Community Health Workers) were recruited to collect information from uncovered areas.

A list of all married women aged 18–49 years living areas was prepared by LHWs and CHWs and a unique code was assigned to each woman. A total of 51,690 women were recorded by the LHWs and CHWs, residing in the study area. Among the listed women, 40,952 (79%) were provided with services by LHWs, and 10,738 (21%) resided in the areas of CHWs. During household visits, LHWs and CHWs registered pregnant women, and followed them up to 42 days after delivery. This process of registration and follow-up continued for the entire study duration. Women delivering in last month of the project were followed until the end of the month.

A list of all public and private health facilities situated in the study area was obtained from the local District Health Authority. Nineteen public health facilities that fulfilled the inclusion criteria, i.e. providing antenatal, delivery, postnatal or newborn care, were included into the study. A copy of monthly reports of selected public health facilities was obtained from the Provincial cell of the District Health Information System. Ten private health facilities that provide maternity services (inpatient and outpatient), or pediatric services (both inpatient and outpatient) were included in the study. A focal person was nominated by the District Health Officer to collect monthly reports from the private hospitals which were submitted to the District Health Office.

Mother and Child Health cards were used to capture data on pregnancies, births and deaths from both LHW’s and CHW’s areas. These are currently used by the LHWs to capture data from pregnant women on all previous pregnancies, prenatal care, medical conditions, delivery and pregnancy outcomes including baby alive or dead, birth weight, sex, and newborn complications. Data from public and private health facilities were obtained through the *DHIS Monthly Reporting Forms (DHIS-21 and 22)*. These forms are currently in use by the Health Department to collect data from the public health facilities and currently report data on services provided by the health facility. The reporting form for secondary health facilities differs from that of the Primary Health Care Facility Monthly Report form in that it has additional inpatient and laboratory data.

LHWs and CHWs were trained on the process of selecting eligible women ages 18 to 49 years, enlisting them, and taking informed written consents in their respective areas. CHWs recruited for the research project were provided with an additional 2 days training on correct completion of the Mother and Child Health Card, monthly report forms, and referral procedures. Of 73 CHWs selected for the research project, 61 (84%) attended the initial training session, and those who could not attend were trained in subsequent training sessions at their respective health facilities. One day training was provided to staff engaged for data collection from the public and private hospitals on correct completion of DHIS monthly reports, and collection of the reports from the selected private healthcare providers and hospitals, and selected public hospitals.

### Analysis

Descriptive statistics were used to summarize demographic and mortality data. Proportions were used for categorical variables, and means were used for continuous variables. For maternal mortality, cohort mortality using pregnancy as a denominator could be calculated, but aggregate mortality rates using live births as a denominator were used, as these are more familiar and usual. For neonatal mortality the denominator is live births. For perinatal mortality, total births (alive or still), was used as a denominator. Period mortality rates in the study area, calculated from the data collected by the enhanced surveillance system for 2015/16, were compared with mortality rates for the same area, calculated from routine LHWs aggregated data from 1.5 years prior (2014–15) to the enhanced surveillance system, to estimate under-enumeration of death rates. The prior LHWs data for 1.5 years was used due to its availability and to maximize the numbers of births and deaths for the comparison. The neonatal mortality for the routine LHWs data prior to the enhanced surveillance system is estimated from the proportion of early neonatal deaths (< 7 days) in the study area calculated by the enhanced surveillance system. A comparison is also made between LHW data (only) collected by the enhanced surveillance with the prior 1.5 years LHWs data to assess the improved completeness of recording by LHW, with an exclusion of effects of improved coverage in the CHW areas. Rates of stillbirths, early neonatal deaths, neonatal deaths and perinatal deaths by LHWs and CHWs areas were calculated and chi-squared tests and *p*-values used to determine whether there were any significant differences in these rates. Mortality rates were based on 12 months of recorded data for 2015/16. Maternal mortality ratio was measured using the number of deaths of a woman dying during pregnancy or within 42 days (6 weeks) of termination of pregnancy from pregnancy related causes per 10^5^ live births in the same year. Neonatal mortality rate: newborn death within the first 28 days of life (0–27 days) per 10^3^ live births in 1 year. Early neonatal mortality rate: newborn death within the first 7 days of life (0–6 days) per 10^3^ live births in 1 year. Stillbirth rate: baby born without signs of life after 28 weeks of gestation per 10^3^ births. Perinatal mortality rate: stillbirths and early neonatal deaths combined per 10^3^ births in 1 year.

National and Provincial maternal and neonatal mortality rates reported by DHSs and international agencies (WHO, UNICEF, UNFPA and World Bank) during 1990 to 2016 were plotted to compare mortality rates calculated by the enhanced surveillance system and LHWs data for the sub-district. Statistical analysis was performed using SPSS Version 24 (SPSS Inc., Chicago, IL, USA) and Manual 10 for demographic estimation using Excel spreadsheets [16].

To assess the similarity of the population in the study area to the Province population, comparisons of total fertility rate [16], general fertility rates [16], mean Body Mass Index (BMI) as weight (kg)/[height (m)]^2^ and sex ratio at birth in the study area were compared with the Province as estimated by the DHS 2012/13. No DHS survey was conducted after 2013 (Table 1). The only statistically significant difference (*p* < 0.001) observed was between the proportion who were a healthy weight (BMI 18.5–24.9) and overweight (BMI 25.0–29.9). The Table 5 in Appendix shows characteristics of the study population in greater detail.

**Table 1:** Population characteristics of Tehsil Havelian (study area) and Khyber Pakhtunkhwa Province, Pakistan

Characteristics	Study area^a^ 2015/16	Province^b^ DHS sample 2012/13	*p*-value^c^
Fertility			
Annual live births	7273	350	
Married WRA	51,690^d^	2695^e^	
General Fertility Rate^f^	141	130	0.168
Total Fertility Rate^g^ (95% CI)	4.3 (4.0–4.6)	3.9 (3.7–4.2)	ns
Body Mass Index			
Median	24.5	25.4	
Underweight (> 18.5)	522 (6.9)	54 (5.8)	0.203
Normal (18.5 - 24.9), n (%)	3603 (47.8)	390 (41.9)	< 0.001
Overweight (25.0 - 29.9), n (%)	1997 (26.5)	313 (33.7)	< 0.001
Obese (≥30.0), n (%)	1422 (18.8)	173 (18.6)	0.856
Total women, n (%)	7544 (100)	930 (100)	
Sex ratio at birth^h^	106	107	0.538
Males	3655	1532	
Females	3857	1436	

*Abbreviations:* *WRA* Women of Reproductive Age, *DHS* Demographic Health Survey, *CI* Confidence Intervals, *ns* not significant based on 95% CI

^a^Date collected by enhanced surveillance system (June 2015 to May 2016)

^b^DHS survey data 2012/13 for Khyber Pakhtunkhwa

^c^from chi-square

^d^age 18–49 years

^e^age 15–49 years

^f^General Fertility Rate is annual live births/10^3^ Married WRA

^g^Total Fertility Rate per woman

^h^Sex ratio is for total births (for provincial sample, total births include 10 years data i.e. from 2002 to 2012)

## Results

Higher maternal mortality, perinatal mortality and neonatal mortality rates were observed through enhanced surveillance compared to mortality rates estimated by the routine monitoring system. Integration of data from various sources to identify maternal, perinatal and neonatal deaths and extending coverage to previously uncovered areas, improved the enumeration of births and deaths and provided accurate mortality rates in the study area. A small proportion (4%) of the study population living in very remote areas could not be completely covered by the enhanced surveillance system for the entire duration of the study.

### Maternal mortality

MMR of the LHWs area was lower at 226/10^5^ live births (95% CI; 124–379) compared with MMR of 370/10^5^ live births (95% CI; 101–948) in CHWs area calculated from the enhanced surveillance system (*p* = 0.38) in 2015/16 (Table 2). MMR of 226/10^5^ live births in LHWs of areas calculated by the enhanced surveillance system was higher than MMR of 180/10^5^ live births estimated from previous 1.5 years routine LHWs data indicating improved completeness (Table 3). The maternal mortality ratio in the study area calculated from the enhanced surveillance system data was 247/10^5^ live births (95% CI; 147–391) for 2015/16, compared with the MMR of 180/10^5^ live births (95% CI; 101–297) estimated from previous routine LHWs data (*p* = 0.36) for 2014/15 (Table 4). Based on these data, maternal mortality in the study area was underestimated by 27% by routine surveillance in 2014/15.

**Table 2:** Maternal mortality, stillbirth, perinatal and neonatal mortality in LHWs and CHWs areas in Tehsil Havelian, District Abbottabad, Pakistan enhanced surveillance 2015/16^d^

Events	Total			LHWs Areas			CHWs Area			*p*-value^e^	CHW: LHW
	n	Rate	95% CI	n	Rate	95% CI	n	Rate	95% CI		
Maternal Mortality^a^	18	247	147–391	14	226	124–379	4	370	101–948	0.380	
Neonatal Mortality^b^	290	40	35–44	247	40	35–45	43	40	28–51	0.992	1.00
Early Neonatal Mortality^b^	215	30	26–33	184	30	25–34	31	29	19–39	0.857	1.03
Perinatal Mortality^c^	454	60	55–66	366	57	52–64	88	77	62–93	**0.009**	1.35
Stillbirths^c^	239	32	28–36	182	29	24–33	57	50	37–63	**< 0.001**	1.72
Denominators (n)											
Total births	7512			6375			1137				
Live births	7273			6193			1080				

*Abbreviations*: *CI* Confidence Interval

^a^per 10^5^ live births

^b^per 10^3^ live births

^c^per 10^3^ total births

^d^June 2015 to May 2016

^e^derived from chi-square

Bold: significant at *p* < 0.05

**Table 3:** Maternal, perinatal, and neonatal mortality in Lady Health Workers’ (LHW) areas (only) from the enhanced surveillance system in Tehsil Havelian compared to prior routinely collected LHW data

Events	Tehsil Havelian - LHWs areas only						*p*-value^h^	Under-enumeration^i^ (%)
	Routine LHW data^a^ 2014/15^f^ 1.5 years (1 year)			LHW data: Enhanced Surveillance System 2015/16^e^ 1 year				
	n^g^	Rate	95% CI	n	Rate	95% CI		
Maternal Mortality^b^	15	180	101–297	14	226	124–379	0.541	20
	(11)	(188)	(94–336)				0.647	17
Neonatal Mortality^d^	170	20	17–23	247	40	35–45	**< 0.001**	50
	(128)	(22)	(18–26)				**(< 0.001)**	45
Early Neonatal Mortality^d^	126	15	13–18	184	30	25–34	**< 0.001**	50
	(95)	(16)	(13–19)				**(< 0.001)**	46
Perinatal Mortality^c^	401	47	42–51	366	57	52–64	**0.003**	18
	(305)	(50)	(45–56)				(*0.080*)	12
Stillbirths^c^	275	32	28–36	182	29	24–33	0.227	−10
	(210)	(35)	(30–39)				(*0.052*)	−19
Denominators (n)								
Total births	8599 (6062)			6375				
Live births	8324 (5852)			6193				

Bold: significant at *p* < 0.05. *Italics*: marginally significant. 95% CI: normal approximation of the binomial. Stillbirth: dead baby ≥28 weeks of pregnancy per total births; Abortions, dead fetus < 28 weeks of pregnancy; Early Neonatal Mortality, newborn death (0-6 days) per live births; Neonatal Mortality, newborn death (< 28 days) per live births; Perinatal Mortality: stillbirths plus early neonatal deaths per total births

*CI* Confidence Interval

^a^prior to enhanced surveillance system

^b^per 10^5^ live births. Poisson distribution used to calculate 95% confidence intervals

^c^per 10^3^ total births, normal approximation of binomial counts used to calculate 95% CI

^d^per 10^3^ live births

^e^1 June 2015–31 May 2016

^f^1 January 2014–31 May 2015

^g^in brackets is 1 year data

^h^derived from chi-square

^i^by LHW data, negative under enumeration = over enumeration

**Table 4:** Maternal, perinatal, and neonatal mortality from the enhanced surveillance system in Tehsil Havelian compared to previous routinely collected data from Lady Health Workers only

Events	Tehsil Havelian (study area)						*p*-value^g^	Under-enumeration (%)
	Routinely collected data (LHW only)^a^ 2014-15^f^			Enhanced Surveillance System 2015/16^e^				
	n	Rate	95% CI	n	Rate	95% CI		
Maternal Mortality^b^	15	180	101 – 297	18	247	147 - 391	0.362	27
Neonatal Mortality^d^	170	20	17 – 23	290	40	35 - 44	**< 0.001**	50
Early Neonatal Mortality^d^	126	15	13 – 18	215	30	26 - 33	**< 0.001**	50
Perinatal Mortality^c^	401	47	42 – 51	454	60	55 - 66	**< 0.001**	22
Stillbirths^c^	275	32	28 – 36	239	32	28 - 36	0.953	0
Denominators (n)								
Total births	8599			7512				
Live births	8324			7273				

Bold: significant at *p* < 0.05. 95% CI: normal approximation of the binomial. Stillbirth: dead baby ≥28 weeks of pregnancy per total births; Abortions, dead fetus < 28 weeks of pregnancy; Early Neonatal Mortality, newborn death (0–6 days) per live births; Neonatal Mortality, newborn death (< 28) per live births; Perinatal Mortality: stillbirths plus early neonatal deaths per total births

*CI* Confidence Interval

^a^Tehsil Havelian (study area), prior to enhanced surveillance system

^b^per 10^5^ live births (Poisson distribution used to calculate 95% confidence intervals)

^c^per 10^3^ total births (normal approximation of binomial counts used to calculate 95% CI)

^d^per 10^3^ live births

^e^1 June 2015 to 31 May 2016

^f^1 January 2014-31 May 2015

^g^derived from chi-square

### Neonatal mortality

The neonatal mortality rate (NMR) of 40/10^3^ live births (95% CI; 35–44) calculated by enhanced surveillance system for 2015/16 was higher than NMR of 20/10^3^ live births (95% CI; 17–23) in the study areas estimated from the previous routine LHWs data (*p* < 0.001) for 2014/15. This is a 50% underestimation of neonatal mortality compared with the enhanced surveillance in the next year. The NMRs of LHWs and CHWs area in 2015/16 were similar with no statistically significant differences (Table 2). A significantly higher NMR in LHWs areas was observed with a NMR of 40/10^3^ live births (95% CI; 35–45) calculated by the enhanced surveillance system, compared with the previous NMR of 20/10^3^ live births (95% CI; 17–23) estimated by routine LHWs data (*p* < 0.001) indicating improved completeness (Table 3).

### Early neonatal mortality

ENMR was similar in LHWs and CHW areas: 30/10^3^ versus 31/10^3^ live births (*p*-value not significant) (Table 2). A significantly higher ENMR of 30/10^3^ live births (95% CI; 25–34) in LHWs areas calculated by the enhanced surveillance system was observed compared to NMR of 15/10^3^ live births (95% CI; 13–18) estimated by routine LHWs data (*p* < 0.001) indicating improved completeness (Table 3). Early neonatal mortality rates (ENMR) of 30/10^3^ live births (95% CI; 26–33) calculated by enhanced surveillance system for 2015/16 was higher (*p* < 0.001) than ENMR of 15/10^3^ live births (95% CI; 13–18) estimated by previous 1.5 years routine LHWs data in the study area for 2014/15. The degree of underestimation was estimated at 50% (Table 4).

### Perinatal mortality

From these data perinatal mortality was underestimated by 22% in the study area in 2014/15. The PMR observed in LHWs areas was lower at 57/10^3^ births (95% CI; 52–64), compared to 77/10^3^ births (95% CI; 62–93) in CHWs areas (*p* = 0.009) in 2015/16 (Table 2). A significantly higher (*p* = 0.003) PMR of 57/10^3^ births (95% CI; 52–64) was observed in LHWs areas calculated by the enhanced surveillance system compared with PMR of 47/10^3^ births (95% CI; 42–51) estimated by routine LHWs data indicating improved completeness. Perinatal mortality rate (PMR) calculated from the enhanced surveillance system was 60/10^3^ births (95% CI; 55–66) for 2015/16, which was significantly higher (*p* < 0.001) than the PMR of 47/10^3^ births (95% CI; 42–51) for the study area estimated from prior routine LHWs data for 2014/15 (Table 4).

### Stillbirths

The stillbirth rate (SBR) of 32/10^3^ births (95% CI; 28–36) calculated by enhanced surveillance system for 2015/16 was the same as the SBR of 32/10^3^ births (95% CI; 28–36) prior to the enhanced surveillance system in the study area for 2014/15. There was a statistically significant lower (*p* < 0.001) SBR in LHWs areas compared to CHWs areas (29 versus 50/10^3^ births) for 2015/16 (Table 2). No difference in SBR was observed in LHWs areas before and after the enhanced surveillance system (Table 3).

### Sex difference

Rates of stillbirth (38 versus 25/10^3^ births), early neonatal mortality (36 versus 23/10^3^ live births, perinatal mortality (73 versus 48/10^3^ births) and neonatal mortality (42 versus 38/10^3^ live births) were all higher in males than females in 2015/16. This difference was statistically significant for all rates except neonatal mortality (Table 6 in Appendix).

**Fig. 3:**
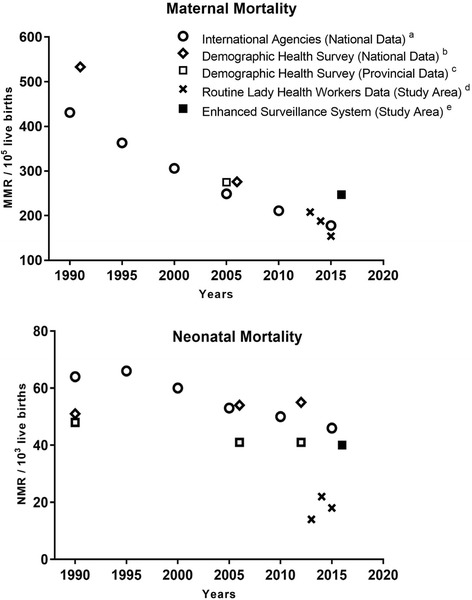
Maternal and neonatal mortality in the study area, Tehsil Havelian, Abbottabad, compared to Pakistan provincial and national estimates. ^a^WHO, UNICEF, UNFPA and World Bank modeled estimates maternal and neonatal mortality for 1990, 1995, 2000, 2005, 2010 and 2015; ^b^Demographic Health Survey national data for maternal mortality for 1991 and 2006 and for neonatal mortality for 1991, 2006 and 2012; ^c^Demographic Health Survey provincial data for maternal mortality for 2006 (data point offset to 2005 due to overlapping points) and for neonatal mortality for 1991, 2006 and 2012; ^d^Routine Lady Health Workers data for maternal and neonatal mortality for 2013, 2014 and 2015; ^e^Enhanced surveillance system data for maternal and neonatal mortality for 2015/2016. Abbreviations: MMR, Maternal Mortality Ratio; NMR, Neonatal Mortality Rate

A national MMR of 178/10^5^ live births was reported by the international agencies in 2015, whereas the only available MMR of 275/10^5^ live births in the province was estimated by a DHS conducted in 2006-07 (Fig. 3).

A national NMR of 46/10^3^ live births for 2015 was reported by the international agencies compared to a NMR of 40/10^3^ live births calculated by the enhanced surveillance system. The DHS for 2012–13 reported national and provincial NMR of 55/10^3^ live births and 41/10^3^ live births, respectively (Fig. 3).

## Discussion

This study demonstrates the under-reporting of maternal and neonatal mortality rates in the study area compared to the previous 1.5 years; maternal mortality was under-estimated by 27% and neonatal mortality by 50%. The mortality rates in the study area calculated from the enhanced surveillance system were higher than those estimated from the previous routine LHWs data. This study provides accurate maternal, perinatal and neonatal mortality rates by establishing an enhanced surveillance system that captured births and deaths from 96% of the study population, through multiple data sources, including data collection from the public and private health facilities and extended community coverage, in a rural area of Khyber Pakhtunkhwa province, Pakistan. During enhanced surveillance 2015/16 higher maternal, neonatal and perinatal rates were found in CHWs areas subject to extended coverage than in the LHWs areas reflecting the more rural and remote character of the previously uncovered areas. The inclusion of the CHW areas increased coverage of women by 20%, but this could not be maintained for 4% because of difficulties associated with remoteness. Comparison of the LHWs data 2015/16 with the previous LHWs data shows that enumeration by LHWs improved with enhancement of surveillance, quite apart from expansion of coverage to new areas. Improvement in maternal, neonatal and perinatal mortality was due to improved completeness as a result of data collection on births and deaths from all sources including community, public and private health facilities, and increased coverage to CHW areas with higher maternal and neonatal mortality.

The surveillance system successfully integrated birth and death data from available routine health information system and extended the surveillance system to the areas and health facilities from where previously no birth or death data were reported. The robust enhanced surveillance system provided sufficient evidence of underestimation of mortality rates in the study area through before and after comparison of mortality rates separated by 1.5 years. The comparison of the study area with itself is closely related in time (1 year), with no change in socioeconomic status and health system, and no disaster, or epidemic diseases or civil disturbance over the comparison period. Although a higher maternal mortality was calculated using the enhanced surveillance system data in the study area compared to routine health information systems, the number of maternal deaths were not sufficient to demonstrate a statistically significant difference in maternal mortality rates. This is a consequence of the short duration of the study, and the small number of maternal deaths.

This is the first study in Pakistan to include private health facilities in a maternal and perinatal surveillance system. The proportion of deliveries reported by private health facilities was 11% of the total births in the study area. This proportion is less than the 2012/13 DHS Provincial statistics of 24% of births [5]. Possible reasons for the discrepancy may include lower affordability for private hospitals in Tehsil Havelian than that for the Province, or there may be under-reporting of births by the private hospitals for various reasons. Nevertheless, the likelihood of missing any birth or death that occurs at private health facilities is much less, because 96% of the population in the study area was covered either by the LHWs or by the CHWs during the enhanced surveillance in 2015/16.

Estimates of MMR in Pakistan suggest a decline from 431/10^5^ live births in 1990 to 178/10^5^ live births in 2015. Although a substantially higher maternal mortality was observed by the enhanced surveillance system in the study area of Tehsil Havelian (247/10^5^ live births), compared to the national MMR of 178/10^5^ estimated by the international agencies (World Bank, WHO, UNICEF, UNFPA) for the year 2015, the Provincial MMR of Khyber Pakhtunkhwa estimated by 2006/07 DHS was consistent with the enhanced surveillance system at 275/10^5^ live births. A study by Sathar reported an estimated national MMR of 220/10^5^ live births in 2012. The study also estimated Khyber Pakhtunkhwa Province MMR of 423/10^5^ live births in 2001, 275/10^5^ live births in 2006, and 206/10^5^ live births in 2012 [17]. A population-based prospective study on active surveillance of pregnancies and their outcomes conducted in six countries, including Pakistan, during 2010-2012 reported a MMR of 313/10^5^ live births, in a rural District of Sindh province [18], similar to MMR reported by DHS 2006/07. A retrospective study of facility-based maternal mortality which collected data for 10 years (2002–2012) in a tertiary care hospital of Khyber Pakhtunkhwa province (Pakistan) reported a MMR of 772/10^5^ live births [19], but this may be affected by referral of complicated cases. Under-enumeration of maternal and neonatal deaths by the LHWs was noted in a study in Lahore (Punjab Province) in 2010, that reported underreporting of maternal and infant deaths by LHW due to fear of not maintaining adequate performance indicators [9]. Verification of LHWs reports showed 92.5% correctly reported maternal death, while 5% underreported and 2.5% over reported maternal deaths [9].

The neonatal mortality in Pakistan changed little during the past two decades according to survey data from the DHS, and the enhanced surveillance neonatal mortality rate is consistent with national and provincial estimates around 2015, but much higher that the LHW data (Fig. 3). The neonatal mortality rate reported by Pakistan DHS 1990/91 was 51/10^3^ live births (1986–1990), Pakistan DHS 2006/07 (2002–2006) was 54/10^3^ live births and Pakistan DHS 2012/13(2008–2012) was 55/10^3^ live births [5]. A non-significant decrease in neonatal mortality was reported by DHS 2012/13 in Khyber Pakhtunkhwa, from 48/10^3^ live births in 1990 to 41/10^3^ live births in 2012 (Fig. 3) [5]. A population-based prospective study reported a neonatal mortality of 50/10^3^ live births, in rural District of Sindh province during 2010–2012 [18].

The stillbirth morality rate estimated from the previous 1.5 years routine LHWs data was higher (32/10^3^ births) compared with the stillbirth rate estimated from the LHWs data collected by the enhanced surveillance system (29/10^3^ births). The over-enumeration of stillbirths by LHWs could be due to their under-enumeration of early neonatal mortality. An international review in 2006 found that a live birth may be recorded as a stillbirth if the baby died immediately after birth, because of various reasons including inadequate knowledge, avoidance of blame, fear of extra work, or poor assessment for the signs of life [20]. A study conducted in 2011/13 [21] reported stillbirth rates of 50/10^3^ births in District Thatta (rural district), Sindh Province of Pakistan. Another prospective study in Sindh Province reported similar higher rates of stillbirths (66/10^3^ births) in 2003 [22]. These rates were higher than calculated from our enhanced surveillance system, as well as that estimated by Pakistan DHS 2012/13.

It is worth noting that neonatal mortality (40/10^3^ live births), early neonatal mortality (30/10^3^ live births), stillbirths (32/10^3^ births) and perinatal mortality (60/10^3^ births) in the study area in 2015, is similar to Provincial neonatal mortality (41/10^3^ live births), early neonatal mortality (33/10^3^ live births), stillbirths (31/10^3^ births) and perinatal mortality (63/10^3^ births) estimated by the DHS in 2012/2013 [5]. However, interpretation needs to take account the differences in methods and time period of 8 years between our study in 2015/16 and those estimated by the DHS 2012/13, and the differences between the Tehsil Havelian and the entire province.

Adolescent women are considered high risk for adverse pregnancy outcomes. A recent population-based prospective study conducted from 2010 to 2013 in six low and middle-income countries including Pakistan, reported a higher rate of maternal, neonatal and perinatal mortality among women aged 15–19 years compared to women age ≥ 20 years [23]. This study does not include pregnant women aged < 18 years as the sample size would be insufficient for subgroup analyses, and extended consent would be required from parents and additional approval from research ethics committees.

Higher rates of stillbirth, early neonatal, neonatal and perinatal mortality among males than females are consistent with the Pakistan DHS 2012/13 that reported higher neonatal mortality in males compared with females in Pakistan. An analysis using data from the Pakistan Demographic Health Survey 2006/07 reported a statistically significant hazard ratio of 1.57 for neonatal mortality in males compared to female neonates [24]. This is also consistent with international statistics where neonatal and infant mortality in males are reported to be higher than females which provide further validation for the study [25]. Reasons for this higher mortality in males are explained by biological factors, including a higher risk of respiratory syndrome (related to late maturity), infectious diseases, congenital malformations of the urogenital system in males, and fetal growth retardation [26–28]. Population characteristics of the study population were similar to the provincial population in terms of total fertility rate, general fertility rates, body mass index and sex ratio at birth (Male/Female) [5]. Hence the results of the study likely reflect the Provincial population.

The Pakistan DHSs estimated maternal, perinatal and neonatal mortality only at the national and provincial level, which may mask the district or sub-district variations in mortality rates. The need to access district and sub-district data is also reported by a South African study in 2016 [29], which emphasizes the need to use disaggregated data at the sub-district level for equitable resource allocation and targeting the areas in need. A study on a vital events surveillance system in India estimated causes of maternal and neonatal deaths in 2012 using CHWs and supported the application of targeted community-based interventions that resulted in a significant reduction in neonatal mortality [30].

The LHW Program, having 70% coverage of national population, provides an opportunity to measure accurate mortality rates at the sub-district level if coverage is enhanced to capture the entire population. The enhanced surveillance system demonstrated that the birth and death data reported by LHWs, CHWs (for enhanced coverage), community midwives, health care facilities (public and private), and the routine health information system (DHIS), can be reconciled to provide accurate and timely mortality rates at a district and sub-district level. This could be used to strengthen the healthcare delivery system through the application of area-specific and cause-specific targeted healthcare interventions and improving the coverage of current health care program in Pakistan. This surveillance system can enable health managers to utilize resources more efficiently and target them to the area most in need, thus have a maximum impact of the targeted intervention in the reduction of mortality rates.

Following decentralization of services in Pakistan (18th Amendment of the Constitution) in 2010, it is imperative to have accurate maternal, perinatal and neonatal mortality rates at the district and sub-district level. In addition, the local government ordinance highlights the need to empower local governments and improve the governance by decentralized decision-making [8, 31].

Accurate and timely data on mortality is required to monitor progress, implement health interventions and to evaluate health programs at national and sub-national levels [32, 33]. A global assessment of civil registration and vital statistics reported most South Asian countries, including Pakistan, have weak vital civil registration with inadequate coverage and poor quality data on deaths and causes of deaths [33]. In these circumstances, maternal, perinatal and neonatal mortality data are obtained from household censuses, Demographic Health Surveys [34], Multiple Indicator Cluster Surveys [35] and reproductive age mortality surveys [36], employing direct death inquiry of household members over a retrospective period, and/or indirect methods such as children ever-born and children surviving, and orphanhood and widowhood [37, 38] questions in national and/or sub-national surveys [39]. However, these sources have various limitations, including underestimation of maternal deaths and requirement of large sample sizes [40–42]. Although a population census may be a better approach to measuring mortality rates than surveys, there are issues with data quality and omission of up to 50% of deaths in population censuses has been reported [42].

A small proportion (4%) of the study population living in very remote areas could not be completely covered by the surveillance system for the entire duration of the study. Eighteen CHWs recruited for these areas registered 2599 women of reproductive age (18–49 years), ten left during the first month, and eight afterwards, mostly due to the arduous nature of the work. However, 37 births and no deaths were recorded by the CHWs prior to their resignation. These data were not included in analyses.

Further research is needed to demonstrate the feasibility of using this enhanced surveillance system that integrates births and deaths data from all possible sources for application of area-specific and cause-specific interventions with measurement of the impact of the reduction in mortality rates. This is particularly required in districts with low community coverage by LHWs and Midwives. Opportunities should also be explored to link births and deaths captured by routine health information systems with civil registration authorities to strengthening civil registration and vital statistics. Research is also needed to measure the effects of adequate surveillance on Mother and Child Health Programs and expected reductions in MMR and NMR.

## Conclusion

A surveillance system that triangulates birth and death data from all health information sources, extends coverage and follows all pregnancies for the outcome, is needed to generate accurate mortality estimates at a district and sub-district level. A decline in maternal mortality in Pakistan was reported by the international agencies based on DHS. However, when maternal mortality was measured in a district through near complete enumeration of births and deaths by following all pregnancies in a defined population, a higher maternal mortality was observed. Similarly, perinatal, early neonatal and neonatal mortality in the study area was significantly higher than that estimated from the available data. There is no surveillance system in Pakistan that provides accurate and timely maternal, perinatal and neonatal mortality data at the district and sub-district level. This makes the problem less visible to policymakers and program managers.

A robust surveillance system that is capable of providing district and sub-district mortality rates in order to target areas with higher mortality rates is essential, and thus can lead Pakistan towards achieving the Sustainable Development Goals.

## Appendix

**Table 5:** Characteristics of the Study population, Tehsil Havelian, District Abbottabad, 2015/16^a^

Characteristics	Category (n)	%
Age, yr. (*n* = 7572)		
Median	28	
Range	18–48	
18–19	175	2.3
20–34	6174	81.5
35–49	1223	16.1
Parity (*n* = 7572)		
Median	1	
Range	0–11	
0	2052	27.1
1–4	4935	65.2
≥ 5	585	7.7
Child < 2 yr. (*n* = 7572)		
Yes	1213	16
No	6359	84
Height, cm (*n* = 7551)		
Median	157	
Range	107–196	
≤ 142.24	2275	30.1
> 142.24	5276	69.9
Weight, kg (*n* = 7565)		
Median	56	
Range	35–95	
≤ 45	639	8.4
49–79	6741	89.0
≥ 80	185	2.4
Body Mass Index (*n* = 7544)		
Median	24	
Range	13–56	
Underweight (< 18.5)	522	6.9
Normal (18.5 – < 25)	3603	47.8
Overweight (25 – < 30)	1997	26.5
Obese (≥ 30.0)	1422	18.8

^a^1 June 2015 to 31 May 2016

**Table 6:** Stillbirths, early neonatal, neonatal and perinatal mortality, by gender, in the study area, Tehsil Havelian, District Abbottabad, Pakistan 2015/16^c^

Events	Total			Females			Males			*p*-value^d^	Male: Female
	n	Rate	95% CI	n	Rate	95% CI	n	Rate	95% CI		
Stillbirths^a^	239	32	28–35	92	25	20–30	147	38	32–44	**0.001**	1.58
Early NM^b^	215	30	26–33	82	23	18–02	133	36	30–42	**0.003**	1.57
NM^b^	290	40	35–44	134	38	31–04	156	42	36–49	0.329	1.11
PM^a^	454	60	55–66	174	48	41–55	280	73	64–81	**< 0.001**	1.52
Denominators (n)											
Total births	7512			3655			3857				
Live births	7273			3566			3707				

*Abbreviations*: *CI* Confidence Interval, *NM* Neonatal Mortality, *PM* Perinatal Mortality

^a^per 1000 total births

^b^per 1000 live births

^c^June 2015 to May 2016

^d^derived from chi-square

Bold: significant at *p* < 0.05

## Abbreviations

BMI: Body mass index
CHW: Community Health Worker
DHIS: District Health Information System
DHS: Demographic Health Surveys
ENMR: Early neonatal mortality rate
GFR: General fertility rate
LHW: Lady health worker
MDGs: Millennium Development Goals
MMR: Maternal mortality ratio
NM: Neonatal mortality
NMR: Neonatal mortality rate
PM: Perinatal mortality
PMR: Perinatal mortality rate
SBR: Stillbirth rate
TFR: Total fertility rate
WHO: World Health Organization
WRA: Women of reproductive age
